# HiCmapTools: a tool to access HiC contact maps

**DOI:** 10.1186/s12859-022-04589-y

**Published:** 2022-02-10

**Authors:** Jia-Ming Chang, Yi-Fu Weng, Wei-Ting Chang, Fu-An Lin, Giacomo Cavalli

**Affiliations:** 1 Department of Computer Science, National Chengchi University, 11605 Taipei City, Taiwan; 2 Institute of Human Genetics, CNRS and University of Montpellier, Montpellier, France

**Keywords:** Hi-C, Topologically Associating Domains (TADs), 3D genome, Juicer, hicpipe

## Abstract

**Background:**

With the development of HiC technology, more and more HiC sequencing data have been produced. Although there are dozens of packages that can turn sequencing data into contact maps, there is no appropriate tool to query contact maps in order to extract biological information from HiC datasets.

**Results:**

We present HiCmapTools, a tool for biologists to efficiently calculate and analyze HiC maps. The complete program provides multi-query modes and analysis tools. We have validated its utility on two real biological questions: TAD loop and TAD intra-density.

**Conclusions:**

HiCmapTools supports seven access options so that biologists can quantify contact frequency of the interest sites. The tool has been implemented in C++ and *R* and is freely available at https://github.com/changlabtw/hicmaptools and documented at https://hicmaptools.readthedocs.io/.

**Supplementary Information:**

The online version contains supplementary material available at 10.1186/s12859-022-04589-y.

## Background

With the invention of the microscope, researchers gained a preliminary understanding of the chromosome's tertiary structure. However, it was difficult to gain a more global picture, that is, until the development of chromosome conformation capture (3C) [1] and its variations 4C [2], 5C [3], and HiC [4], which have made available spatial information on the whole genome. There are now many HiC pipelines available [5, 6]. However, there is no suitable tool to access HiC map results except for visualization (Table 1), that is, no systematic way to extract HiC contact information for a specific query. For example, given the list of CTCF binding sites, a custom script is needed to compute all pairwise contacts between them. Therefore, we have developed *HiCmapTools*, which helps biologists efficiently query HiC maps and perform permutation tests. It supports seven query modes and attempts to cover the most frequent needs of biologists who use HiC to study chromatin contacts and their putative function.

**Table 1:** Comparison between HiCmapTools and other current tools applied to HiC sequencing datasets

Function	HiCmapTools	HiCPro [7]	Juicer [8], Juicebox [9]	gcMapExplorer [10]
Generate HiC map	x	o	o	x
Visualization	x	x	o	o
Format transformation	x	o	o	o
Extra submap	o	x	dump	x
Query HiC map	o	MAKE_VIEWPOINTS.PY*	x	x

^*^Generates a BED profile from a specified viewpoint (similar to the -bait query mode of HicmapTools)

## Implementation

HiCmapTools is implemented in C+ + , which facilitates using common programming data structures and functions from the Standard Template Library (STL). Users input HiC maps in either *.hic* format generated by Juicer [8] or bin-contact pair files following hicpipe [11, 12]. The input contact map is stored as a hash structure using pair bins as keys. The size of the bin is specified by the user (-in_hic_resol for .*hic*) or depends on the input file (bin-contact pair files). A query is binned into a corresponding key based on its position to facilitate efficient extraction of contact frequency via STL hash operation (*O*(1) for lookup). Also, we measure the significance of the extracted frequencies using permutation tests which rank the frequency among random samples. The usage of the query mode and random test are explained below.

### Query mode

**Fig. 1:**
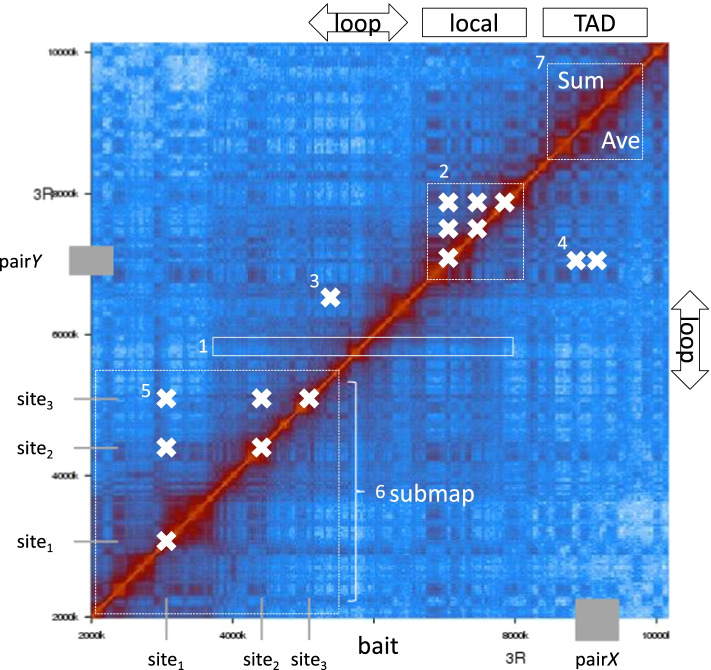
Illustration of query modes. Numbers indicate the corresponding query modes. HiC data is *Drosophila* chr3R:2000k..10000k [14]

We use seven query modes to meet the needs of biologists, as illustrated in Fig. 1. The query input is expected to be in BED format, in which each line is considered as an individual query. Sample query files are available at https://hicmaptools.readthedocs.io/en/latest/format.html#query-file.

1. bait: calculate average contacts from downstream to upstream (controlled by -ner_bin) of a position of interest (white rectangle). For example, biologists can measure the average contact frequency around a PRE binding site.
2. local: list all contacts inside an interval (white cross). All contacts inside a gene body can be extracted by querying specific gene loci.
3. loop: contact frequency between two ends of a loop. As an example query, biologists can test whether gene looping exists [13] by calculating the contact frequency of its promoter with the transcription termination site. A gene of interest is listed as one row in a BED file.
4. pair: contacts between a pair of regions (contact between regions *X* and *Y*, white crosses). For instance, contact frequencies between a gene promoter and an enhancer are extracted by querying their positions.
5. sites: contacts between specific sites (contacts between three sites, including diagonal). As an example, given the list of chromatin insulator sites, HiCmapTools calculates all pairwise contacts among these sites, such that users can check whether any pair of binding sites interact with each other.
6. submap: sub contact map of regions of interest. The HiC map is stored efficiently by keeping only selected regions (i.e., a region containing long-range contacts between two loci such as the *Drosophila* Antp-C and the BX-C).
7. TAD: sum and average of contacts within specific TAD regions (white dashed square at the top right of Fig. 1). Biologists can quantify chromatin compaction within a TAD by measuring the average intra-TAD contact frequency. This might be used to compare different TADs of interest.

### Permutation test

The biological meaning of the extracted contact frequency is assessed by the probability that a given frequency occurs by chance. We approximate the distribution of the null hypothesis by shuffling query positions. That is, we generate alternative queries by randomly shifting the query loci around the same chromosome multiple times (default = 100 times, controlled by the -random option). Then, we perform the same query mode for the alternative queries and calculate their contact frequencies. Finally, the query's contact frequency is evaluated as its ranking among the sampled frequencies.

### bait query example

Here, one is interested in whether there is local contact enrichment around specific loci, such as the Ubx gene in the BX-C locus. Then, we perform a -bait query where a 30k map is used, up/downstream is 150kb (= 30k x 5bins) with 100 permutation tests.

hicmaptools -in_map fly_30k.n_contact -in_bin fly_30k.cbins -bait Ubx.bed -near_bin 5 -random 100 -output Ubxt-bait.tsv

**Fig. 2:**
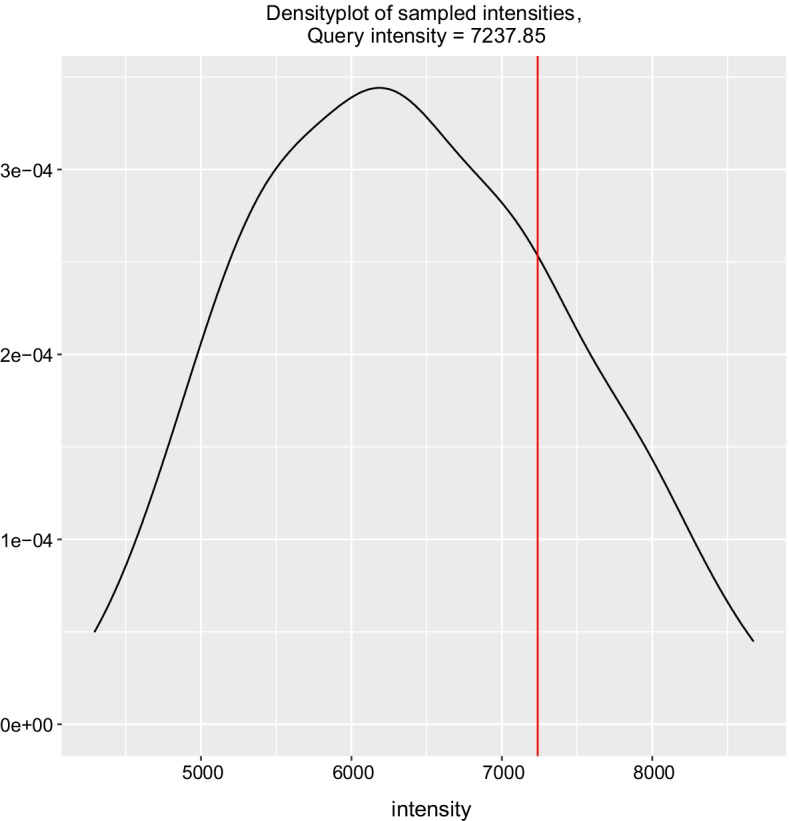
Plot of the permutation test. Density shows the frequency of the permutation test. Query frequency is highlighted as a red vertical line

Two outputs are produced: *Ubxt-bait.tsv* and *Ubxt-bait_random_1.txt*. The former contains the contact frequency of the query (for example “7237.85”) and the average of the sampled frequencies (for example “6363.51”) with comparison provided as ratio (for example “1.14”) and rank (for example “top 19%”). The latter provides each sampled frequency, including that of the query (the second line), where the suffix “_1” indicates one query entry in the input file. Output details are available online at https://hicmaptools.readthedocs.io/en/latest/format.html#output. We provide *tools/visualPermutationTest.R*, a *R* script to visualize the query's output against the distribution of the random samples (Fig. 2).

Rscript visualPermutationTest.R Ubxt-bait_random_1.txt UbxPermuTest.pdf

Besides *Drosophila*, HiCmapTools could handle other species. We test whether there is local contact enrichment around the *Tox* gene in mouse T Cell. The HiC map, *D0_mm10.hic*, is available at https://doi.org/10.6084/m9.figshare.18586106 instead of GitHub due to the size limit.

hicmaptools -in_hic D0_mm10.hic -bait Tox_mm10.bed -random 0 -output Tox_bait_D0.txt

## Results

HiC data has shown chromosome structure to be formed by 3D folding of a higher-order unit, the topologically associating domain (TAD) [14–16]. In *Drosophila*, TADs can be further classified into four epiTADs—*active*, *null*, *PcG,* and *HP1—*based on ChIP-seq binding profiles [14]. Although a substantial proportion of TADs form loops, with loop bases frequently located at the TAD boundaries in mammals [17, 18], few examples of TAD loop structure have been reported in the fly genome [19]. These loops generally involve Polycomb binding sites, but whether any preference exists regarding epiTADs has not been systematically addressed. We selected two related experiments to determine whether HiCmapTools can help biologists address these questions.

We approached the question by applying HiCmapTools on high-resolution *Drosophila* HiC data (1 kb, merged embryo from GSE 34,453, 61,471, 94,115, and 99,105, “Availability of data and materials”). There are 1257 TADs with 548 active, 469 null, 136 PcG, and 104 HP1 TADs (Additional file 1).

### TAD loop

We calculate the contact frequency between two boundaries of the TADs via the *loop* query mode:

hicmaptools -in_map fly.bimap -in_bin fly.bins -loop epiTAD.bed -output resLoop.tsv

**Fig. 3:**
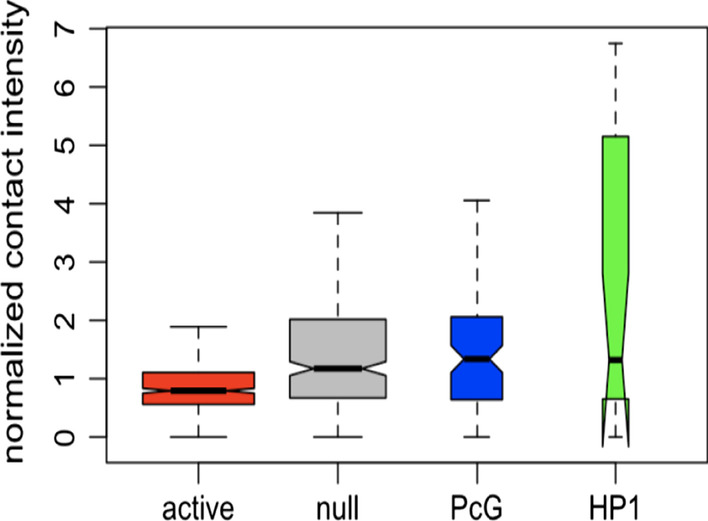
Boxplot of loop frequency regarding epiTADs

Interestingly, PcG TADs show strong loop frequency, whereas active TADs have the lowest frequency (Fig. 3, raw data in Additional file 2). Therefore, we assume that PcG TADs form a loop structure with high probability [20].

### TAD intra-density

Furthermore, we check whether intra-TAD contact frequencies also show differences across epi-classes via the *TAD* query mode:

hicmaptools -in_map fly.bimap -in_bin fly.bins -TAD epiTAD.bed -output resTADs.tsv

**Fig. 4:**
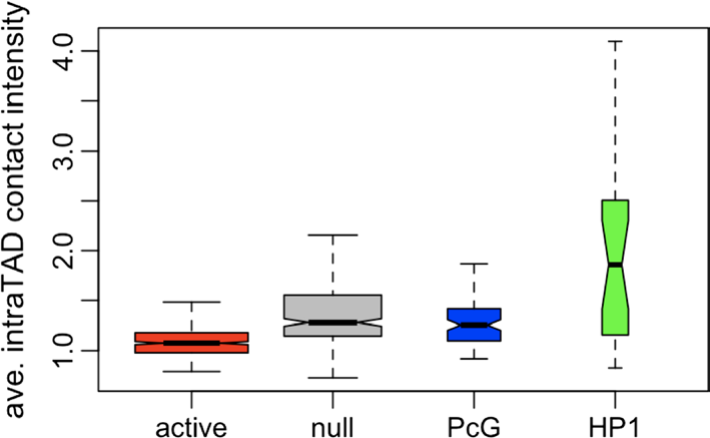
Boxplot of average intra-TAD contact frequencies regarding epiTADs

HP1 TADs show a higher average intra-TAD contact density than the other three classes, whereas active TADs show the lowest contact density (Fig. 4, raw data in Additional file 3). This is in agreement with previous studies which reported that active chromatin domains present a weaker inside contact density than inactive domains, PcG, and null epiTADs [16].

### Running time benchmark

Each query command generally completes within minutes. However, this varies based on the input scale, especially HiC maps. We conducted the following tests (Table 2) based on different input map resolutions and file formats on a personal desktop (4 GHz CPU, 32 GB memory).

**Table 2:** HiCmapTools runtimes for different query modes, map resolutions, and map formats of the fly map (dm3), where runtimes are recorded in user mode

Resolution	Format	Size	Query	Per.Test	Time (sec)	ps
30 k	bin-contact/text	67 MB	bait	100	6.23	Section “bait query example”
1 k	bin-contact/binary	2.4 GB	loop	0	11.34	Section “TAD loop”
				100	11.45	
			TAD	0	11.32	Section “TAD intra-density”
				100	14.30	
	bin-contact/text	3.2 GB	loop	0	345.62	
				100	525.14	
			TAD	0	344.43	
				100	525.54	

The most time-consuming step of HiCmapTools is parsing the map and turning it into a hash structure, especially for text format. The running time increases from 6.23 s to 345.62/344.43 s when the map size increases from 67 MB to 3.2 GB in text format. A text HiC map can be saved as a binary format via the genBinMap command and loaded without parsing.  This speeds up the running time by a factor of around 30 (345.62 → 11.34, 344.43 → 11.32).

## Conclusion

We present a C+ + package that provides an efficient way to query HiC maps. HiCmapTools supports seven access options so that biologists can quantify the contact frequency of the interest sites. Furthermore, the frequency probability is estimated based on a null hypothesis that shuffles the query position. Finally, the frequency is visualized as an output plot: a vertical line in the density plot of the random samples. The authors will continue to develop new functions for comparative HiCs to pursue HiC quantitative analysis.

## Availability and requirements

- Project name: HiCmapTools
- Project home page: https://github.com/changlabtw/hicmaptools
- Project document page: https://hicmaptools.readthedocs.io/
- Operating system(s): platform-independent
- Programming language: C+ + and R
- Other requirements: none
- License: GNU GPL
- Any restrictions to use by non-academics: license needed

## Supplementary Information

**Additional file 1.** The list of epiTADs in bed format where the fourth column notes the epi-class: 1- active-red, 2- null-gray, 3-PcG-blue and 4-HP1-green.

**Additional file 2.** *Bash* and *R* scripts for the experiment of “3.1 TAD loop”.

**Additional file 3.** *Bash* and *R* scripts for the experiment of “3.2 TAD intra-density”.

## Data Availability

The merged *Drosophila* embryo HiC contact map in bin-contact pair format: Bin: https://doi.org/10.6084/m9.figshare.14730918. Contact: https://doi.org/10.6084/m9.figshare.6965576.

## Abbreviation

TAD: Topologicallyy Associating Domain
