# Supplementary figures and images for: HiCmapTools: a tool to access HiC contact maps
Source: BMC Bioinformatics. 2022 Feb 10;23:64. doi: 10.1186/s12859-022-04589-y (PMC8832839; doi:10.1186/s12859-022-04589-y)

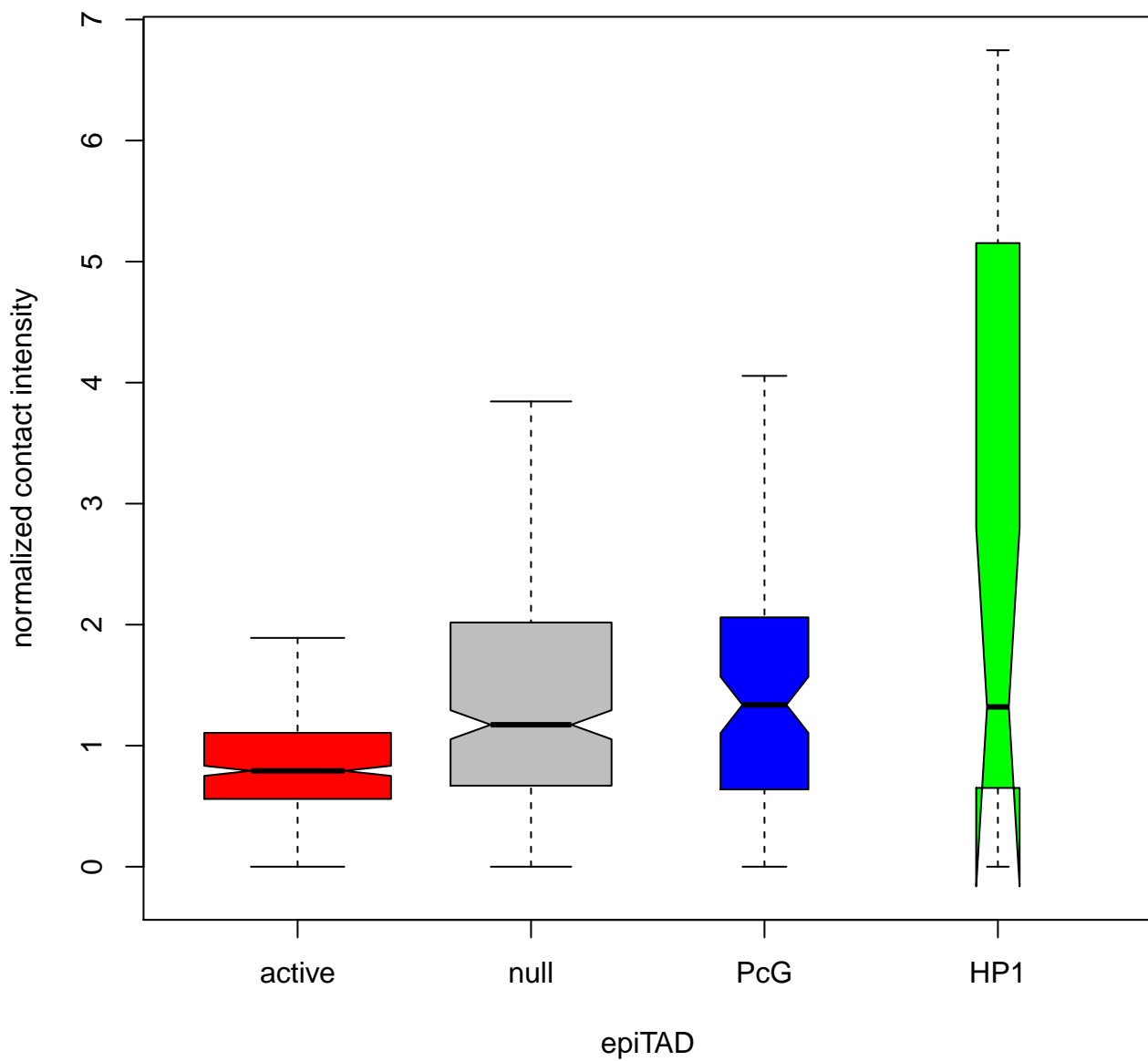

Supplement: Supplementary file 2 — Additional file 2. Bash and R scripts for the experiment of “3.1 TAD loop”. [file 12859_2022_4589_MOESM2_ESM.zip › loopIntensity4epiTAD.pdf]

TADintra\_cm\_10k\_top5.tsv

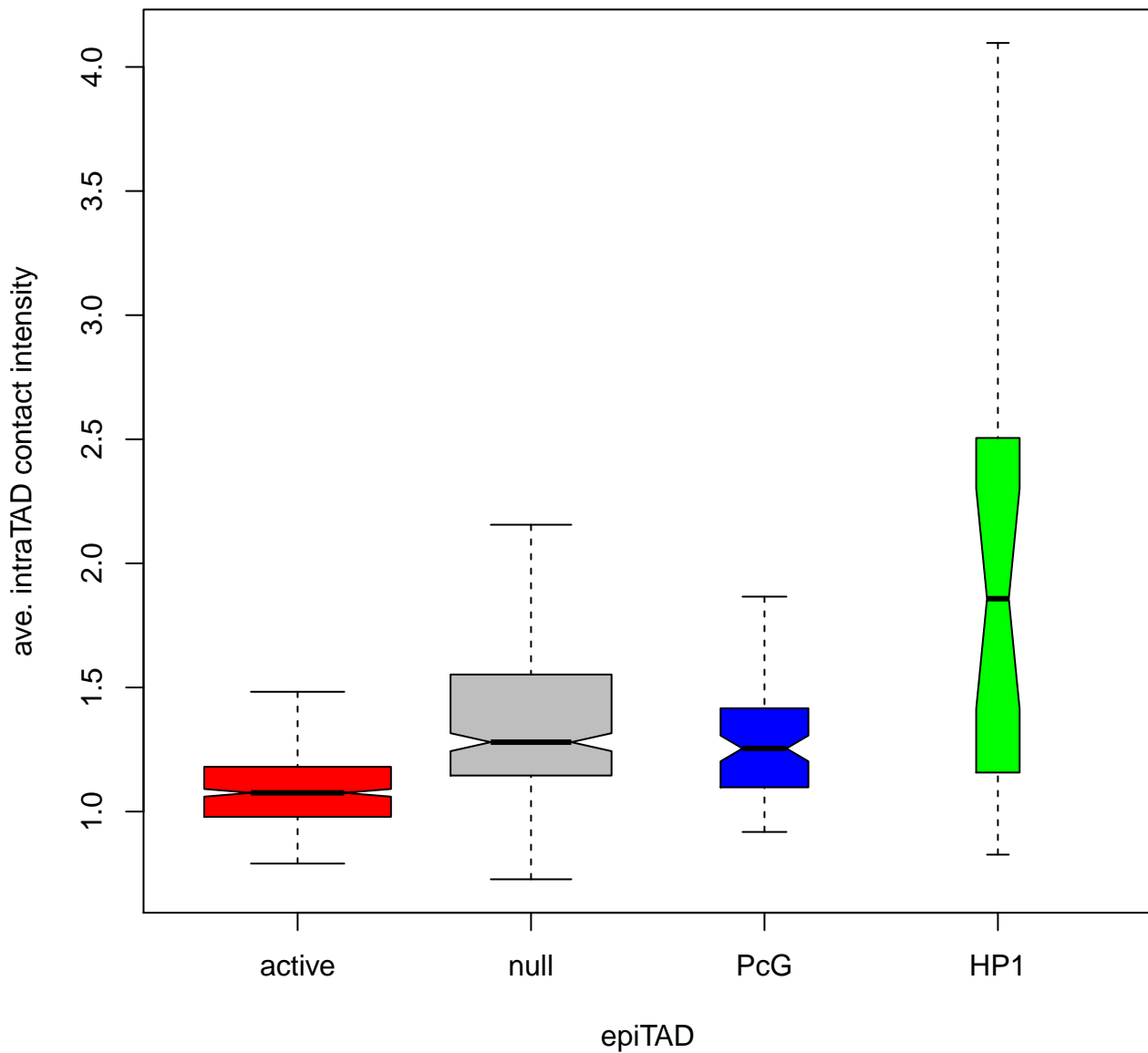

Supplement: Supplementary file 3 — Additional file 3. Bash and R scripts for the experiment of “3.2 TAD intra-density”. [file 12859_2022_4589_MOESM3_ESM.zip › intraIntensity4epiTAD.pdf]
